# Editorial: Many roads to anammox

**DOI:** 10.3389/fbioe.2022.963008

**Published:** 2022-12-21

**Authors:** Sitong Liu, Qilin Wang, Kenji Furukawa

**Affiliations:** ^1^ College of Environmental Sciences and Engineering, Peking University, Beijing, China; ^2^ Centre for Technology in Water and Wastewater, School of Civil and Environmental Engineering, University of Technology Sydney, Ultimo, NSW, Australia; ^3^ Graduate School of Science and Technology, Kumamoto University, Kumamoto, Japan

**Keywords:** anammox, nitrification, nitrogen removal, nitrogen cycle, wastewater treatment

Anaerobic ammonium oxidation (Anammox) has been proposed as one of the most promising alternatives to achieve an energy-efficient urban wastewater treatment plant (WWTP). Over the past few decades, anammox-based processes have been successfully implemented in sidestream treatment. However, the evolution of anammox bacteria, cell biology, and new anammox microorganisms is still not clear. The engineering applications of anammox at low temperatures and achieving a promising way to supply nitrite for anammox in mainstream treatments are still limited. Additionally, the function and contribution of anammox bacteria to the nitrogen cycles of different ecosystems need to be determined. In order to promote the application of anammox and to further explore the connection between anammox and other microorganisms, our Research Topic contains articles on the studies of anammox and nitrogen removal. This Research Topic includes five articles that are innovative and interesting and report achievements in the process of anammox and efficient biological nitrogen removal strategies from the perspectives of metabolomics, metagenomics, and microbial ecology.

Anammox is an important process in the nitrogen cycle, widely occurring in terrestrial, freshwater, and marine ecosystems. Partial nitritation, as a promising way to support nitrite for anammox, has received a lot of attention in this Research Topic. In order to explore the diversity and function of bacteria involved in nitrogen removal, Xu et al.analyzed the microbiota compositions of different kinds of sludge at a full-scale partial nitritation-anammox (PN/A) plant. This investigation uncovered the partition mechanism of anammox and nitrifiers in a full-scale PN/A plant. It is the first assessment of the microbiota compositions of three different types of sludge in a full-scale sidestream PN/A plant, providing a contribution for guiding the replication and development of full-scale PN/A systems. In this PN/A plant, biofilm sludge exhibited a higher anammox functional activity, and suspended sludge presented a higher partial nitritation activity. The highest numbers of anammox bacteria were detected in biofilm sludge, and ammonia-oxidizing bacteria (AOB) were mainly present in suspended sludge. Candidatus *Brocadia* was the dominant bacteria in biofilm sludge. *Nitrosomonas* was the main AOB in suspended sludge.

Since the PN/A process has low treatment costs, it has been widely applied in sewage treatment. However, the nitritation process is extremely sensitive to variations in dissolved oxygen (DO) and substrate concentrations. In order to provide a stable supply of nitrite for mainstream wastewater treatment, Zhang et al. operated five sequencing batch reactors with different carrier materials, including a non-woven carrier, polyurethane carrier, brucite-nonwoven carrier, zeolite-nonwoven carrier, and sepiolite-nonwoven carrier. The sepiolite carrier played a crucial role in the inhibition of nitrite-oxidizing bacteria (NOB). The numbers of *Nitrospira* and *Nitrobacter* in sepiolite-nonwoven carrier were lower than in other materials. The sepiolite-nonwoven composite carrier can effectively improve the partial nitritation process, which is significantly helpful for the development of PN/A for mainstream sewage treatment.

Various other materials and elements influence nitrogen metabolic bacteria. For example, heavy metals, at high concentrations, inhibited anammox microorganisms. However, they also require trace amounts of different types heavy-metal elements to maintain their activities and growth. Isaka et al. determined the limiting effects of Ni(II), Zn(II), and Se(VI) in an anammox reactor during the start-up period. Both Ni(II) and Se(VI) were not necessary in the anammox process. Zn(II) seriously influenced and limited the anammox process during start-up.

As the name of this Research Topic “Many Roads to Anammox” suggests, the articles in our Research Topic not only focus on nitrogen metabolic bacteria, but also on microalgae. Compared with other traditional sewage treatments, the algal-bacterial method is more useful for improving energy savings. Yang et al. demonstrated the PN/A process, with *Chlorella sorokiniana* suppling optimal oxygen. The authors successfully started the algal-intensified PN/A system using algammox biofilm. In this photosequencing system, *C. sorokiniana*, AOB, and anammox coexisted. The dominant nitrogen metabolic bacteria in this algal-intensified PN/A system included Candidatus *Brocadia* (anammox) and *Nitrosomonas* (AOB). *Denitratisoma* (denitrifier), *Nitrospira*, and *Nitrobacter* (NOB) were inhibited. This study contributes to the development and application of algal-bacterial systems for PN/A.

Since the relationships between algae and bacteria are complicated, Wu et al. analyzed the relative contribution of quorum sensing by using indole-3-acetic acid (IAA) in an algal-bacterial reactor during nitrogen removal. The reactor included *Chlorella* which exhibited a higher nitrogen removal rate (NRR) than *Phormidium*; the NRR profile was similar to the numbers profile of the quorum sensing-related gene. *Chlorella* was the dominant quorum sensing-related algae. *Pseudomonas*, *Hydrogenophaga*, and *Zoogloea* were the dominant quorum sensing-related bacteria. The quorum sensing process influenced the algal-bacterial correlation. This study hinted at the significance of the quorum sensing relationship between algae and bacteria in this interkingdom interaction during sewage treatment.

Innovative experiments related to, and applications of anammox are reported in this Research Topic. The findings in these articles are helpful for further deciphering the mechanisms of anammox. Furthermore, this Research Topic could be helpful for enhancing planning management strategies for sewage treatment to improve nitrogen removal, providing a theoretical basis for understanding the anammox process.

